# Temperature Acclimation Ability by an Oceanic Sea Skater, *Halobatesgermanus*, Inhabiting the Tropical Pacific Ocean

**DOI:** 10.3390/insects9030090

**Published:** 2018-07-24

**Authors:** Takahiro Furuki, Hiroki Fujita, Mitsuru Nakajo, Tetsuo Harada

**Affiliations:** 1 Laboratory of Environmental Physiology, Graduate School of Integrated Arts and Sciences, Kochi University, Kochi 780-8520, Japan; tfuruki9269@docomo.ne.jp (T.F.); gg02@kochi-u.ac.jp (H.F.); 2 Laboratory of Science Education, Graduate School of Integrated Arts and Sciences, Kochi University, Kochi 780-8520, Japan; mit-na@kochi-u.ac.jp

**Keywords:** Heteroptera, oceanic sea skaters, *Halobates germanus*, temperature acclimation, cool hardiness, global warming

## Abstract

Temperature acclimation and heat shock experiments were performed on adult oceanic skaters, *Halobates germanus*, inhabiting the tropical Pacific Ocean. Acclimation for 10 or 24 h to 25 °C or 28 °C promoted significantly lower cool coma temperatures by specimens than acclimation to 31 °C. After heat shock by exposure to the relatively moderate temperature of 32.5 °C for 12 h, 52.9% or 61.1%% of specimens died in the 24 h period following acclimation at 28 °C or 31 °C, respectively, whereas all survived when there was no experience of heat shock. The average cool coma temperature was 14 to 17 °C in the specimens which had suffered no heat shock, whereas it was much higher (22 to 23 °C) in specimens that had suffered heat shock. The lower survival rate and the higher cool coma temperature can be attributed to damage suffered by exposure to 32.5 °C. The upper limit of the surface water temperature in the tropical ocean (15° N to 15° S) is currently around 30 to 31 °C, and *Halobates* appear to have no experience in 32 to 33 °C environments. Nevertheless, 32 °C, i.e., a temperaturethat is only slightly higher than 30 to 31 °C, may occur in the future due to global warming. This species may develop resistance to 32 to 33 °C in the near future.

## 1. Introduction

### 1.1. Heat Shock, Hardening and Acclimation in Ectotherms

Bowler [1] defined the terms, ‘acclimation’, ‘heat shock’, and ‘hardening’ in regard to temperature response based on thermal physiology in ectotherms. (1) Acclimation means “physiological or behavioral changes occurring within an organism and itis caused by experimentally induced stressful changes in particular climatic factors”. (2) Heat shock means “a rapid, short acting molecular process associated with the synthesis of several families of heat shock proteins (hsps) of different molecular weights, such syntheses can be elicited as a result of acute short sub-lethal heat injury.” (3) Hardening means “a quick, transitory adaptation to an extreme temperature (hot or cold) that followed brief exposure at a sub-lethal temperature including hsps also related to enhancement of cold hardiness” [2].

### 1.2. Effects of Higher Temperature Acclimation

High temperature acclimation induces a high metabolic rate [3]. Mosquitoes, *Culex pipiens,* were reared in a full factorial design at 18 or 26 °C as larvae and adults, and then critical thermal maxima (CTmax) and metabolic rate-temperature (MR-T) relationships were determined for all treatments. CTmax was positively affected by both larval and adult acclimation treatments. MR-T slope (higher temperature exposure induced high CO_2_ production) was significantly affected only by adult treatment, with a higher angle of the slope at 18 °C than that at 26 °C. These results demonstrate that larval acclimation effects can alter adult phenotypes in a species whose life cycle includes two drastically different environments: an aquatic and a terrestrial stage.

Higher temperature acclimation induces a subsequent higher metabolic rate [4]. In a Coleopteran species, *Chirodica chalcoptera*, the critical thermal maxima (mean ± s.e.: 41.8 ± 0.2 °C in field-fresh beetles) showed a smaller response (<1 °C change) to temperature acclimation (to 12, 19 and 25 °C for 7 days) than the onset of the critical thermal minima (0.1 ± 0.1, 1.0 ± 0.2 and 2.3 ± 0.2 °C, respectively). The metabolic rate measured at 25 °C was higher in 25 °C-acclimated beetles relative to the field-fresh and 12 °C-acclimated beetles. This result would imply that climate change in these habitats could have an impact on this species, in turn possibly affecting the functioning of the local ecosystem.

### 1.3. Effects of Lower Temperature Acclimation

Acclimation to lower temperature can promote higher performance [5]. In a parasitoid fly, acclimation to 15 °C and 22 °C resulted in a higher survival rate and body mass than acclimation to 30 °C. Overall, relatively low temperature promoted higher performance in this species. Lower temperature acclimation can also enhance mitochondrial function [6]. Five days of acclimation at 15 °C enhanced the ability of mitochondrial function to promote ATP production, and subsequently, a higher survival rate at 4 °C in *Drosophila* flies.

Low temperature acclimation promoted the composition of glycerol phospholipid composition in membranes that function to promote cold hardiness in the fruit fly, *Drosophila melanogaster* [7]. After 60 h in 0 °C, more than 80% of the 15 °C-acclimated flies survived, while none of the 25 °C-acclimated flies survived. Low temperature acclimation was associated with an increased proportion of ethanolamine (from 52.7% to 58.5% in 25 °C-acclimated versus 15 °C-acclimated flies, respectively) at the expense of choline in glycerol phospholipids (GPLs). Cold tolerance, but not heat tolerance, was therefore influenced by pre-imaginal acclimation temperature, and correlated with changes in GPL composition in membranes in adult *D. melanogaster*.

However, lower acclimation ability for upper thermal limit plasticity was observedin another strain of *Drosophila melanogaster* and *D. subobscuris*. A review paper by Sǿrensen et al. [8] showed that in some populations of the fruit fly, plasticity of upper thermal limits is small in magnitude, and evolves slowly. The acclimation ability in these populations is weekly correlated with latitude and environmental heterogeneity. Therefore, plasticity in upper thermal limits is unlikely to effectively buffer effects of global warming for species already close to their upper thermal boundaries. 

### 1.4. Effective Acclimation to Lower and Higher Temperatures

Developmental conditions as temperature acclimations in either direction (i.e., hotter or colder) can show effective adaptation results in the adult stage. For instance, temperature acclimation in the larval stage can modify several characteristics of contents of membrane phospholipids, growth rate, wing size, melanisation, egg size, and locomotion speed in *D. melanogaster* [9].

### 1.5. Objectives of This Study: Temperature Acclimation within a Narrow Temperature Variation of 25 to 31 °C and Heat Shock Effects from a Moderate Temperature of 32.5 °C

Oceanic sea skaters inhabit mainly the tropical sea surface where the temperature is very stable throughout the seasons. Therefore, resistance to lower and higher temperature seems to vary across quite a narrow range. Although the exact values for survival in various environmental temperatures is currently unknown for oceanic sea skaters, it is known that they can only survive within a very limited range of temperatures, i.e., between around 15 to 20 °C to 35 to 40 °C in several oceanic areas [10,11,12,13,14,15,16,17,18,19]. In contrast, a terrestrial freshwater *Gerromorphan* species of semi-aquatic insects exhibits a very wide range for lower and higher temperatures: −3 °C to 43 °C [20,21].

Oceanic sea skaters may suffer abrupt 5 °C decreases in temperature under heavy rain, as well as surface water temperatures ranging from 32.5 °C to 33.0 °C in near future due to global warming. Current global warming trends could increase their chances of being exposed to dramatic temperature increases. Therefore, oceanic sea skaters could be exposed to several changes in temperature ranging from 25 °C to 32.5 °C over periods as short as several hours. We therefore carried out experiments on temperature acclimation and moderate heat shock using *Halobates germanus*.

This species inhabits open oceans near the islands where precipitation is high, and therefore, salinity and temperature fluctuations are also high. We made two working hypotheses: (1) They can acclimate to a moderate temperature within the narrow range of 25 to 31 °C. (2) Even a slightly higher temperature of 32.5 °C would be too severe for their survival, because the surface temperature in the tropical Pacific Ocean varies only narrowly between 29 and 31 °C. To test the two working hypotheses, two experiments were performed in this study. The first was a temperature acclimation experiment to 25 °C, 28 °C and 31 °C. The second was a survival experiment at 32.5 °C.

## 2. Materials and Methods

### 2.1. Samplings

Samplings were performed during the evening in darkness over two days, December 7 and 8, 2016, in the area of 5 to 7° S, 101 to 103° E with a neuston net (6 m long and a diameter of 1.3 m). One set of trials comprised towing the neuston net for 15 mm three or four times on the sea surface, per night (7:00 p.m. to 8:00 p.m.). Two sets of trials in total were performed in total with ship speed of 2.0 knot relative to the sea water from the starboard side of a R/V MIRAI (8687t, cruise MR16-08) owned by the Japan Agency for Marine-earth Science and Technology (JAMSTEC). The square of the surface area that was swept by the neuston net was evaluated as an expression of [flow-meter value × 1.3 m of the width of the neuston net].

### 2.2. Treatment of Specimens after Samplings and before the Experiments

Oceanic sea skaters were trapped in a grey plastic cylindrical bottle fixed at the end of the neuston net. They were paralyzed from the physical shock due to the trailing of the net. Paralyzed sea skaters were transferred onto the surface of a paper towel to respire. Many specimens recovered from a coma due to the physical shock within 20 min. The rate of recovery was 50 to 80% of all individuals trapped in the neuston net. If sea skaters were trapped in the jelly of a jellyfish, the jelly was removed from the body very carefully and quickly by hand so that they could recover from the coma. 

### 2.3. Rearing Methods for Moderate Heat Shock and Acclimation

Many white cube aquaria (30 cm × 30 cm × 40 cm) were used to rear the adults who recovered from the coma. Each aquarium contained ten to thirty adult sea skaters of *Halobates*. Specimens during the temperature acclimations were fed adult flies, *Lucillia illustris*, at a rate of one fly per three adult sea skaters. The flies were removed just before the CCEs. In the first experiment (temperature acclimation experiment), before the CCEs, temperature acclimation was performed for 10 h. Acclimation was performed at 25 ± 0.1, 28 ± 0.1 or 31 ± 0.1 °C. In the second experiment (heat shock experiment), experimental specimens were reared for 12 h at 32.5 °C to induce heat shock, and then were transferred and acclimated for 24 h at 28 °C or 31 °C. The survival rate was calculated at the end of the 24-h temperature acclimation period for both groups. The survival rate was also calculated at the end of the 24-h temperature acclimation at 30 °C for the non-heat shock group as the control. After that, the specimens were used in CCEs. For adults reared in the aquaria in the laboratory, the old food was replaced with fresh food every 6 to 12 h and the seawater in the aquaria was replaced with new seawater three times, at 8:00 a.m., 1:00 p.m. and 6:00 p.m., to prevent the food from polluting the water.

### 2.4. A rearing Setupbefore Cool Coma Experiments

Adult oceanic sea skaters that recovered from the coma were moved and kept on seawater in aquaria set up in a laboratory. The air temperature in the laboratory was kept at 30 ± 2 °C throughout the experiments. The sea skaters were kept under one of two seawater temperature conditions, 30 °C or 32.5 °C (moderate heat shock temperature) for 12 h. After exposure to the respective temperature condition, they were acclimated to 25 °C, 28 °C or 31 °C in the control temperature (30 °C) group, or to 28 °C or 31 °C in the moderate heat shock (32.5 °C) group. Cool coma experiments (CCEs) were performed after the acclimations (described below).

### 2.5. Cool Coma Experiments (CCEs)

Twelve or thirteen adults were moved from the cube aquaria in which they had been kept under the water temperature of 30 °C or 32.5 °C (moderate heat shock for 12 h) to low temperature thermostatic water baths (Thomas: T22LA) (55 cm × 40 cm × 35 cm). The specimens were acclimated at 25 ± 0.1, 28 ± 0.1 or 31 ± 0.1 °C for 10 h for the first experiment before the CCEs. For the second experiment, specimens were acclimated at 28 ± 0.1 or 31 ± 0.1 °C for 24 h before the CCEs.

The water temperature in the baths was gradually decreased by 1 °C every 15 min with an automatic cooling/heating system in the water baths until cool coma (CC) occurred in all experimental specimens.

The temperature at which the CC occurred was recorded as cool coma temperature (CCT) [21]. When CC occurred, the ventral surface of the body stuck to the seawater film, and the ability to skate was lost or abnormal postures on the seawater were observed (such as one leg sinking into the water, being upside-down, or a mid-leg thrust behind and stuck to a hind leg). Gap temperature for cool coma (GTCC) was also calculated as the value of the difference in temperature between the temperature at which specimen was acclimated and CCT.

### 2.6. Statistical Analysis

Analysis of covariance (ANCOVA) was performed to carry out integrated analysis on the effects of the acclimation temperature and moderate heat shock on cool coma. Chi-squared tests were performed between the control (30 °C) group and the moderate heat shock group (32.5 °C) to analyze the survival rate.

## 3. Results

### 3.1. Distribution

In our samplings of *Halobates* (Table 1) inhabiting tropical stations in the eastern Pacific Ocean, we collected 586 individuals in total of two species, *Halobates germanus* and *H. micans*, at two stations, 04°36′–37′ N 137°19–20′ E (Station A) and 01°59′N 02°00′ 138°46′E (Station B). No individuals of *H. sericeus* were collected. The population densities at Stations A and B were about 129,598 individuals/km^2^ and 15,746 individuals/km^2^, respectively. The dominant species inhabiting these stations was *H. germanus*.

The number of individuals collected for 15 min differed considerably between Station A (maximum: 218 specimens) and Station B (minimum: 9 specimens). The large variation in number of specimens collected is consistent with samplings previously performed during the MR06-05 and MR15-04 cruises. This variation suggests that sea skaters may inhabit the sea surface gregariously rather than homogenously in places such as Station A of this cruise in the tropical ocean area. At Stations A and B, 189 and 19 larvae were collected, respectively, and 12 exuviae (skin cast off at molting) were caught in total. Reproductive and growth activity may be very high at the two stations.

### 3.2. Cool Coma Experiments (CCEs)

Many individuals of *H. germanus* collected at Station A completely recovered from the coma caused by physical shock of the neuston net sweeping. They were used in acclimation and moderate heat shock experiments. Cool coma temperatures (CCTs) and gap temperatures for cool coma (GTCC) were measured in this experiment, and ranged from 12.0 °C to 25.6 °C and 2.0 °C to 17.2 °C, respectively. The mean and standard deviation of CCT and GCCT are shown in Table 2.

### 3.3. Acclimation and Moderate Heat Shock Experiments

**Figure 1:**
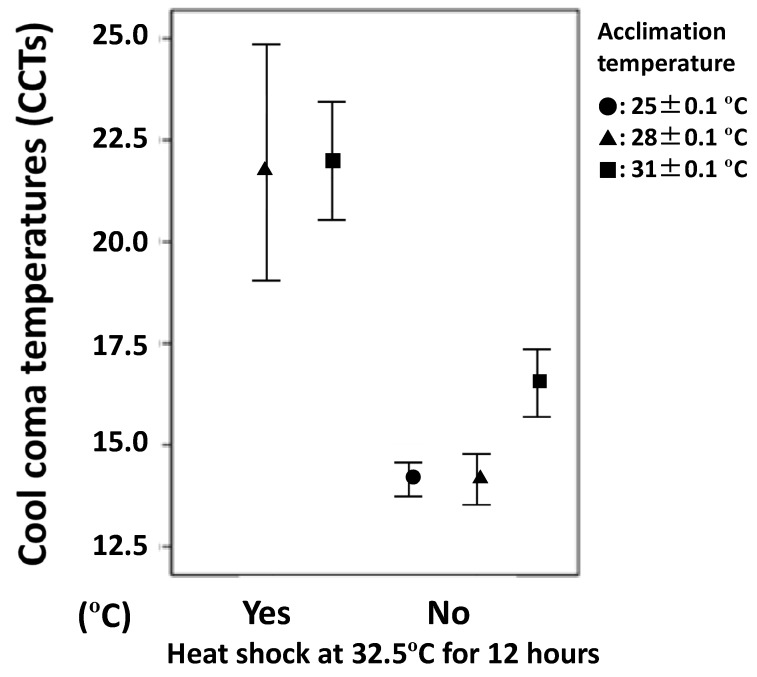
Comparison of cool coma temperature (°C) (CCT: Mean ± 95% confidence value) after heat shock (or not) at 32.5 °C for 12 h and after temperature acclimation for 10 h (or 24 h for heat shock groups) at 25 ± 0.1 °C, 28 ± 0.1 °C, or 31 ± 0.1 °C. Most specimens were adult *H. germanus* collected at Stations A and B during the MR16-08 cruise with the R/V MIRAI.

Specimens acclimated at 25 ± 0.1 and 28 ± 0.1 °C without moderate heat shock showed similar CCTs of 14.5 °C on average (Table 2, Figure 1). However, when they had acclimated at 31 ± 0.1 °C and without moderate heat shock, they showed significantly higher CCTs by 2 °C (Kruskall-Wallis test: χ^2^ − cal = 67.242, *df* = 2, *p* < 0.001) (Table 2, Figure 1). After moderate heat shock, they exhibited CCTs of 21 to 22 °C on average; this was much higher than those without moderate heat shock, at 14 to 17 °C (Table 3, Figure 1).

Specimens that suffered moderate heat shock at 32.5 ± 1.0 °C for 12 h had a significantly lower survival rate of 38 to 47% after 24 h (Table 4). On the other hand, the survival rate after 24 h was more than 90% if there was no heat shock.

### 3.4. Integrated Analysis by ANCOVA

Specimens that were acclimated to 31 °C had higher CCTs than those acclimated to 28 or 25 °C, and damage from heat shock at 32.5 °C for 12 h on CCTs was apparent (ANCOVA on heat shock or no heat shock: *F* = 82.92, *df* = 1, *p* < 0.001, on the acclimation temperature: *F* = 22.25, *df* = 1, *p* < 0.001) (Figure 1). After moderate heat shock at 32.5 °C for 12 h, only 38 to 47% of specimens were still alive after 24 h under 28 °C or 31 °C as the acclimation temperature, whereas the survival rate was more than 0% without the heat shock (Table 3: *F* = 82.922, *df* = 1, *p* < 0.001).

## 4. Discussion

### 4.1. Extreme Week Temperature Tolerance and Acclimation Ability to Moderate Temperature

Insect cold hardiness and effects of acclimation at relatively lower temperature have been studied in depth [22,23]. For example, acclimation at 10 °C for 4 weeks of both larvae in non-diapause and diapause of the Indian meal moth, *Plodia interpunctella* (Lepidoptera: Pyralidae) made mortality rate −5 °C or −10 °C lower compared to non-acclimated larvae [24]. However, this study is a very special case because oceanic sea skaters have an extremely narrow range of temperature tolerance because of narrow environmental temperature variation, i.e., within 29 ± 2 °C, as their habitats of the tropical oceanic surface water temperature within ±15° N or S in the Pacific Ocean. Therefore, the hardiness to lower temperature is very week and the lower coma temperature was only 22 °C or 15 °C on average in oceanic sea skaters. Moreover, an acclimation to “moderate high” temperature of 31 °C which is “maximum” high ambient temperature for tropical oceanic sea skaters can make the cool temperature hardiness weaker, i.e., 2 °C. The results in this study imply that temperature adaptation within a narrow range of 4–5 °C could cause very sensitive responses to small temperature changes. However, in the near future, slightly increased surface temperature in tropical pacific ocean are predicted to be in the order of 0.5 or 1.0 °C. Some individuals are able to change the response to the 31 °C from current negative-one to positive in accordance to the global warming.

### 4.2. Damage to Moderate High Temperature of 32.5 °C and Global Warming

The surface air temperature of the tropical Indian and Pacific Oceans in lower latitudes is very stable at around 25 to 30 °C (surface water: 28 to 31 °C), although it may fluctuate slightly due to weather conditions. For example, it is around 25 °C (surface water: 28 °C) when it rains, but it reaches 30 to 31 °C (surface water: 29 to 31 °C) when the skies are clear (around 29 °C in Lo et al. [25]). However, oceanic sea skaters may not experience surface (water or air) temperatures higher than 33 °C (up to 31 °C even in the tropical water pool area of 90° E to 170° E, 25° S to 15° N: Zhang et al. [26]). The present study demonstrated that *Halobates germanus* was damaged by the moderately high temperature of 32.5 °C in terms of lower survival and lower tolerance. This lower resistance of the species to 32.5 °C is problematic from a biological conservation point of view, as *Halobates* in the tropical ocean may be unable to cope with surface water temperatures reaching 32 °C or 33 °C in the near future due to global warming. Among the very wide variety of temperature adaptation systems in the insect world, oceanic sea skaters have developed a very narrow, sensitive temperature adaptation system, i.e., within 10 °C, due to the very stable temperature characteristics of their habitat. However, they may be able improve their hardiness to upper temperatures of 32 °C or 33 °C in near future due to physiological advances.

## Figures and Tables

**Table 1:** Number of oceanic sea skaters, *Halobates*, collected at locations in the tropical Pacific Ocean on 7–8 December2016,during the science cruise, MR16-08 (N: total number of individuals collected; H.g.: *Halobates germanus*, *H.m.*: *H. micans*; Stat: station number; WT: water temperature (°C); AT: air temperature; L: number of larvae; A: number of adults, E: number of exuviae; EG: number of eggs (on substrates such as polystyrene form); Date: sampling date; Sampling was performed for 15 min; Sa: salinity (‰); S: surface area swept by the neuston net expressed as the value of the flow-meter × 1.3 m^2^ of the width of the neuston net; WS: wind speed (m/s); W: weather; TD: time of day; CS: current speed (m/s); CD: current direction; F: female; M: male. No other species of oceanic sea skaters was collected in this area.

Latitude	Longitude	N	L	A		Hg	Hm	EG	E	Stat	WT	AT	WS	W	CS	Sa	CD	TD	Date	S (×1.3 m^2^)
				F	M															
04°36′ N	137°20′ E	104	35	32	37	97	7	0	1	St.A-1	29.6	28.0	7.7	Cloudy	0.7	32/33.9	95	18:00-15	December 07	
04°36′ N	137°20′ E	115	54	28	33	111	4	0	0	St.A-2	29.6	27.8	7.6	Cloudy	0.8	32/33.9	89	18:24-39	December 07	920.0
04°37′ N	137°20′ E	218	70	73	75	213	5	0	4	St.A-3	29.6	28.1	7.2	Cloudy	0.7	32/33.9	89	18:46~19:01	December 07	768.0
04°37′ N	137°19′ E	101	30	37	34	99	2	0	4	St.A-4	29.6	28.0	6.3	Cloudy	0.7	32	83	19:07-22	December 07	707.0
01°59′ N	138°46′ E	20	10	4	6	19	1	0	2	St.B-1	28.2	24.9	6.8	Cloudy	0.6	32	184	17:59~18:14	December 08	817.0
02°00′ N	138°46′ E	9	4	3	2	9	0	0	1	St.B-2	28.2	24.7	6.6	Cloudy	0.6	32/33.6	182	18:20-35	December 08	819.0
02°00′ N	138°46′ E	19	5	7	7	18	1	0	0	St.B-3	28.2	25.0	8.1	Cloudy	0.6	32/33.6	192	18:41-56	December 08	809.0

**Table 2:** Results of temperature acclimation experiments after acclimation for 10 or 24 h. Cool coma temperatures (CCTs) and gap temperatures for cool coma (GTHCs) were measured in adults *Halobates germanus*. Cool coma experiments were performed after the acclimation to 25 ± 0.1 °C, 28 ± 0.1 °C or 31 ± 0.1 °C. Experiments were performed in December 2016 during the MR16-08 cruise in wet laboratory 2 of the R/V MIRAI.

*Without the Moderate Heat Shock* (30 °C)								
*AcclimationTemperature*	CCT				GTCC			
	Females		Males		Females		Males	
25 ± 0.1 °C	14.33 ± 1.65 (17)		13.79 ± 1.40 (17)		10.67 ± 1.65 (17)		11.21 ± 1.40 (17)	
28 ± 0.1 °C	14.32 ± 2.82 (26)		13.79 ± 1.32 (27)		13.43 ± 2.87 (26)		13.55± 2.03 (27)	
31 ± 0.1 °C ^#^	15.92 ± 1.88 (18)		16.94 ±2.83 (20)		15.08± 0.88 (18)		15.06± 0.62 (20)	
*Two-way ANOVA*	*F-value*	*df*		*p-value*	*F-value*	*df*		*p-value*
*1. Correlation with sex*	0.066	1		0.798	1.417	1		0.236
*2. Correlation with acclimation*	16.950	21		<0.001 ***	53.232	2		<0.001 ***
temperature								
#: 10 h								

# at which specimens were acclimated for 24 h before the Cool Coma Experiments. *: 0.01 < *p* < 0.05; **: 0.001 < *p* < 0.01; ***: *p* < 0.001.

**Table 3:** Results of moderate heat shock experiments after acclimation for 24 h. Cool coma temperatures (CCTs) and gap temperatures for cool coma (GTHCs) were measured in adults *Halobates germanus*. Moderate heat shock was induced for 12 h to 32.5 ± 1.0 °C before acclimation to 28 ± 0.1 °C or 31 ± 0.1 °C. Experiments were performed in December 2016 during the MR16-08 cruise in wet laboratory 2 of the R/V MIRAI.

*After the Moderate Heat Shock* (32.5 °C)				
	CCT		GTCC	
	28 ± 0.1 °C ^+^	31 ± 0.1 °C ^+^	28 ± 0.1 °C ^+^	31 ± 0.1 °C ^+^
*Adults*	21.05 ±3.69 (6)	21.90 ± 1.54 (7)	6.95 ± 3.69 (6)	8.99 ± 1.41 (7)
*Two-way ANOVA*				
*1. Heat shock or not*	*F* = 82.922, *df* = 1, *p* < 0.001 ***		*F* = 115.626, *df* = 1, *p* < 0.001 ***	
*2. Acclimation temp*	*F* = 22.246, *df* = 1, *p* < 0.001 ***		*F* = 90.117, *df* = 1, *p* < 0.001 ***	

+ at which specimens were acclimated for 24 h before the Cool Coma Experiments. *: 0.01 < *p* < 0.05; **: 0.001 < *p* < 0.01; ***: *p* < 0.001.

**Table 4:** The survival rate of specimens just after a 24-h acclimation period when they are exposed to moderate heat shock at 32.5 ± 1.0 °C for 12 h. The survival rate of 50 specimens that did not experience moderate heat shock was 90% (45/50) after acclimation at 30 °C for 24 h.

*Components of Specimens that Acclimated to 28 ± 0.1 °C* or *31 ± 0.1 °C*												
(Number that Survived after 24 h of Acclimation/Initial Number)												
Acclimation temp.	28 ± 0.1 °C						31 ± 0.1 °C					
	*H. germanus*			*H. micans*			*H. germanus*			*H. micans*		
	Adults		5th instars	Adults		5th instars	Adults		5th instars	Adults		5th instars
	F	M		F	M		F	M		F	M	
	2 (5)	4 (9)	0 (1)	2 (2)	0	0	5 (7)	2 (8)	0 (2)	0	0	1
In total	8 (17)						7 (18)					
The rate of survival	47.06 (8/17)						38.89 (7/18)					

χ^2^-test between specimens with and without moderate heat shock at 32.5 °C for 12 h; χ^2^ – cal = 12.437, *df* = 1, *p* < 0.001
